# Identification and validation of glycosylation-related gene signatures for prognostic stratification in sepsis

**DOI:** 10.3389/fimmu.2025.1608082

**Published:** 2025-07-02

**Authors:** Chunyang Li, Haiyan Xue, Lihe Chen, Fengxue Zhu, Jie Li

**Affiliations:** ^1^ Department of Critical Care Medicine, Fuxing Hospital, Capital Medical University, Beijing, China; ^2^ Department of Critical Care Medicine, Peking University People’s Hospital, Beijing, China; ^3^ Bloomsbury Institute of Intensive Care Medicine, Division of Medicine, University College London, London, United Kingdom

**Keywords:** sepsis, prognostic model, glycosylation, immune cell, machine learning

## Abstract

Sepsis is a life-threatening condition caused by a dysregulated host response to infection and is one of the leading causes of morbidity and mortality worldwide. Glycosylation is one of the key modes of protein modification, affecting protein folding, transportation, and localization. Glycosylation patterns are closely related to sepsis, but their specific impact still needs further investigation. This study explored the role of glycosylation-related genes in sepsis through bioinformatics analysis and machine learning, and validated the expression value of the key genes. We identified 38 differentially expressed glycosylation-related genes in sepsis datasets, which divided sepsis patients into two subgroups with different survival outcomes, thus highlighting their prognostic value. Subsequently, we constructed prognostic models using various machine learning methods, classifying patients into high-risk and low-risk groups with significantly different survival rates. We conducted biological analysis of the key genes in the model at the single-cell level and also validated the expression of these key genes in sepsis patient samples. Our study not only enhances the understanding of sepsis glycosylation but also provides a new strategy for clinical diagnosis and prognosis.

## Introduction

Sepsis is a life-threatening condition caused by a dysregulated response to infection, leading to multiple organ dysfunction with high morbidity and mortality rates (1). Despite significant advances in modern medicine, the early diagnosis and effective treatment of sepsis remain major challenges for the global medical community (2, 3). In recent years, the development of molecular biology techniques has provided researchers with a deeper understanding of the pathophysiological mechanisms of sepsis. Large-scale studies in genomics, proteomics, and metabolomics have identified numerous molecular markers and potential therapeutic targets associated with sepsis (4, 5). However, due to the complex and highly heterogeneous nature of sepsis, finding a universal treatment approach seems impractical (6). Therefore, continuous exploration of new disease subtypes and therapeutic targets is crucial for improving the prognosis of sepsis patients.

Among various research directions, protein glycosylation has gained increasing attention as a critical form of post-translational modification due to its pivotal roles in regulating cell recognition, signal transduction, and immune responses (7). Glycosylation refers to the enzymatic process in which sugars (monosaccharides or oligosaccharides) are covalently attached to proteins, lipids, or other organic molecules (8). It is one of the most common and significant post-translational modifications in living organisms (7). The primary types of glycosylation include N-linked glycosylation and O-linked glycosylation (9–11). Beyond these structural functions, glycosylation actively participates in regulating immune responses, including antigen presentation, cytokine signaling, receptor activation, and leukocyte trafficking (12). For instance, alterations in glycosylation can affect the binding affinity of immunoglobulins, the signaling strength of T cell receptors, and the expression of selectins that guide leukocyte migration. In the context of sepsis, aberrant glycosylation patterns have been increasingly recognized as contributors to immune dysregulation. Studies have shown that systemic inflammation during sepsis is associated with altered glycosylation of immunoglobulins, complement proteins, and mucins, which may exacerbate tissue damage and impair host defense (13–15).

However, research on glycosylation in sepsis is currently limited, with many studies not yet providing a holistic view of its multifaceted mechanisms. Current research primarily focuses on specific glycosylation sites or individual forms of glycosylation, limiting the comprehensive understanding of glycosylation’s complex mechanisms in sepsis (14). Additionally, the significant individual variability among sepsis patients and the limited sample sizes in existing studies constrain the generalizability and reproducibility of the findings (14). Furthermore, although the potential impact of glycosylation on clinical outcomes in sepsis is increasingly recognized, its precise molecular mechanisms remain to be thoroughly investigated (14, 15). These limitations underscore the necessity for more systematic and comprehensive studies to elucidate the role and clinical significance of glycosylation in sepsis.

This study utilizes publicly available datasets and clinical samples to conduct large-scale bioinformatics analyses and validate gene expression. It aims to develop a glycosylation-based prognostic model for sepsis using multiple machine learning approaches. Moreover, it seeks to evaluate the diagnostic efficacy of these prognostic factors. Through systematic analysis, the goal is to identify key glycosylation modifications and genes in sepsis. This research not only enhances the understanding of the pathophysiological mechanisms of sepsis but also provides new insights and potential targets for clinical diagnosis and treatment, ultimately improving the prognosis for sepsis patients.

## Materials and methods

### Data acquisition

We obtained 181 glycosylation-related genes from the GlycoGene DataBase (GGDB) database (https://acgg.asia/ggdb2/). We downloaded sepsis-related datasets from the Gene Expression Omnibus (GEO, https://www.ncbi.nlm.nih.gov/geo/) (16): the training cohort GSE65682, which consists of transcriptome sequencing data and corresponding follow-up data from whole blood samples isolated from 42 healthy controls and 760 sepsis subjects. Additionally, the datasets GSE54514 and GSE95233, which include follow-up information, were used to validate the prognostic value of the predictive model. Furthermore, six other sepsis-related datasets (GSE131761, GSE137340, GSE236713, GSE28750, GSE570653, GSE69528) were chose to confirm the diagnostic value of the model. To further investigate the underlying mechanisms, we downloaded GSE1754538 from GEO, which contains single-cell RNA sequencing (scRNA-seq) data from whole blood samples collected from 5 healthy controls and 4 sepsis donors. To validate our findings, GSE176363 was enrolled, which contains 2 healthy controls and 10 sepsis donors. The scRNA-seq data was processed using Seurat for data loading, quality control, and dimensionality reduction. Cell type annotation was performed using the “SingleR” package and cell specific markers were obtained via the FindAllMarkers function to validate the cell type annotation results.

### Study population

We prospectively enrolled 30 patients diagnosed with sepsis and 20 healthy controls as validation dataset. Patients were recruited from the intensive care units of Peking University People’s Hospital from January 2023 to April 2024. The inclusion criteria were based on the Sepsis-3.0 definitions, which require a suspected infection plus a Sequential Organ Failure Assessment (SOFA) score of 2 or higher. Healthy controls were selected from individuals undergoing routine health check-ups who did not have any signs or symptoms of infection or inflammation.

### Identification of hub glycosylation-related genes

We used the “limma” package to identify genes differentially expressed between normal and sepsis patient blood samples (P < 0.05 & |LogFC| > 1) (17). From these, we selected glycosylation-related genes for further analysis. These selected genes underwent functional enrichment analysis using the Metascape database (www.metascape.org/) (18), with P < 0.05 considered significant. Finally, we used univariable Cox regression analysis to identify glycosylation-related genes significantly affecting sepsis prognosis, designating them as Hub-Glys.

### Glycosylation-related patient stratification and survival analysis

We used the “ConsensusClusterPlus” package in R to perform consensus clustering based on Hub-Glys (19). The optimal number of clusters was determined by analyzing the cumulative distribution function (CDF) curve. Subsequently, we used the “prcomp” function in R for principal component analysis (PCA) to confirm the reliability of the clustering. Kaplan-Meier (KM) survival analysis and log-rank tests were conducted using the survival package in R. The survival differences between different groups were analyzed, and the survival status of all patient samples within 25 days was displayed. Immediately after, we identified glycosylation-related differentially expressed genes (Gly-DEGs) through differential analysis between diverse sepsis groups. These candidate genes were then subjected to gene set enrichment analysis (GSEA) using the clusterProfiler 4.8.2 package in R (20), with the Reactome pathway database for functional enrichment. A P value < 0.05 was considered significantly enriched.

### Establishing prognostic model using multiple machine learning approaches

Based on GSE65682 cohort, we used three machine learning algorithms to select the prognostic Gly-DEGs. The “randomForestSRC” package in R was obtained for random survival forest (RSF) analysis (21). An ensemble of 1000 survival trees with default parameters was conducted and the out-of-bag (OOB) error was used to assess model stability. Variables were ranked by minimal depth and variable importance (VIMP). Genes with positive VIMP and consistent presence across trees were retained as candidate prognostic features. We performed CoxBoost algorithm analysis (22), via the CoxBoost package with 10-fold cross-validation to determine the optimal number of boosting steps. Genes with non-zero coefficients were selected. The “glmnet” package for The LASSO regression analysis (23) was conducted using 10-fold cross-validation to determine the optimal λ. Genes with non-zero coefficients at λmin were retained. To select genes for the final prognostic model, we applied LASSO, CoxBoost, and RSF to the Gly-DEGs. Selected genes filtered by at least two of the three algorithms were considered robust predictors and included in the final multivariable Cox regression analysis to establish the Sepsis Risk Score (GRS). This integrative strategy reduced model overfitting and emphasized features supported across multiple selection methods. Based on the GRS, patients were stratified into high and low-risk groups according to the median GRS value, and their prognostic differences were analyzed in GSE65682 and validated in the GSE54514 as well as GSE95233 datasets.

### Meta-analysis

To enhance the clarity of the diagnostic and prognostic value of the GRS, we utilized the “meta” package in R to combine the Odds Ratios (OR) and Hazard Ratios (HR) obtained from multiple studies. Depending on the degree of heterogeneity determined by the I^2^ statistics, we extracted and pooled the data from each study using either a fixed-effect or a random-effects model.

### Assessment of GRS with clinical characteristics and diagnostic efficacy

We utilized the heatmap package to visualize the distribution of clinical characteristics within the GRS groups from the GSE65682 and GSE95233 datasets. Additionally, we employed univariable and multivariable Cox regression to select independent prognostic factors, thereby assessing the independent prognostic performance of the GRS. Furthermore, we evaluated the diagnostic capability of the GRS and its model genes for sepsis by using the “pROC” package to plot Receiver Operating Characteristic (ROC) curves across eight datasets (24).

### Single cell RNA-seq data analysis

We used the “Seurat” R package to preprocess and analyze scRNA-seq data. Normalization was performed using the “NormalizeData” function with the “LogNormalize” method, and the data was converted into a Seurat object. For quality control, we calculated the percentages of mitochondrial and ribosomal genes, excluding cells with fewer than 200 or more than 3000 genes, and those with over 20% ribosomal RNA content. “Harmony” function was applied to correct for potential batch effects. Since all scRNA-seq samples were derived from the same GEO dataset (GSE175453) with unified experimental conditions, no significant batch effect was observed based on UMAP distribution, and batch correction was not applied. We selected the top 3000 variable features using the “FindVariableFeatures” function and standardized the scRNA-seq data. The “ScaleData” and “RunPCA” functions were used to obtain principal components (PCs), followed by dimensionality reduction using Uniform Manifold Approximation and Projection (UMAP). Cell types and subtypes were automatically annotated using the “SingleR” package with the HumanPrimaryCellAtlasData as reference, then further validated using cell-type–specific differential gene expression analysis with FindAllMarkers function. Based on these DEGs and their functional relevance, we confidently validated the major cell types. This data-driven approach provides robust support for cell identity assignment and avoids potential circularity associated with relying solely on predefined marker lists.

### Real-time quantitative polymerase chain reaction

Total RNA was extracted using the TRIzol™ Plus RNA purification kit and reverse transcribed using a reverse transcription kit. RT-QPCR was performed on a AriaMx Real-time PCR System using SYBR^®^ Green real-time PCR master mix. Gene expression was analyzed using the 2ΔΔCt method with the threshold cycle (Ct). Data were normalized to the expression of GAPDH, and reported as expression in controls set to 1. The primer sequences are listed in the **Supplementary Table 1**.

### Statistical analysis

All statistical analyses were conducted using R software environment (version 4.3.1). The Wilcoxon rank-sum test was used to compare differences between two groups. The Kruskal-Wallis test was employed to evaluate differences among more than two groups. Spearman’s correlation was utilized for correlation analysis. A P-value of less than 0.05 was considered statistically significant. The significance levels were denoted as *p < 0.05; **p < 0.01; ***p < 0.001.

## Results

### Identification of glycosylation-related differential genes

**Figure 1:**
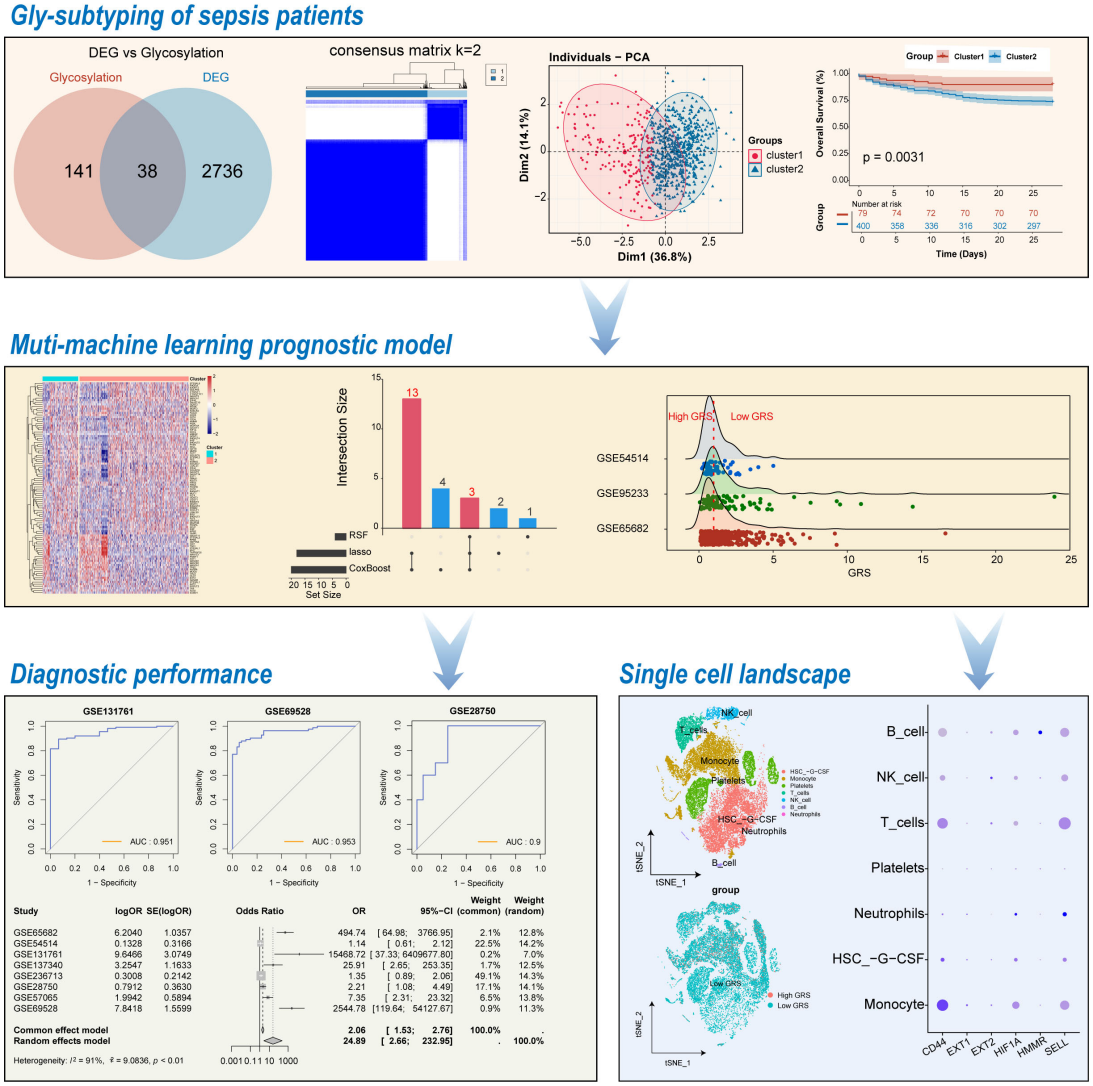
Workflow of this study.

**Figure 2:**
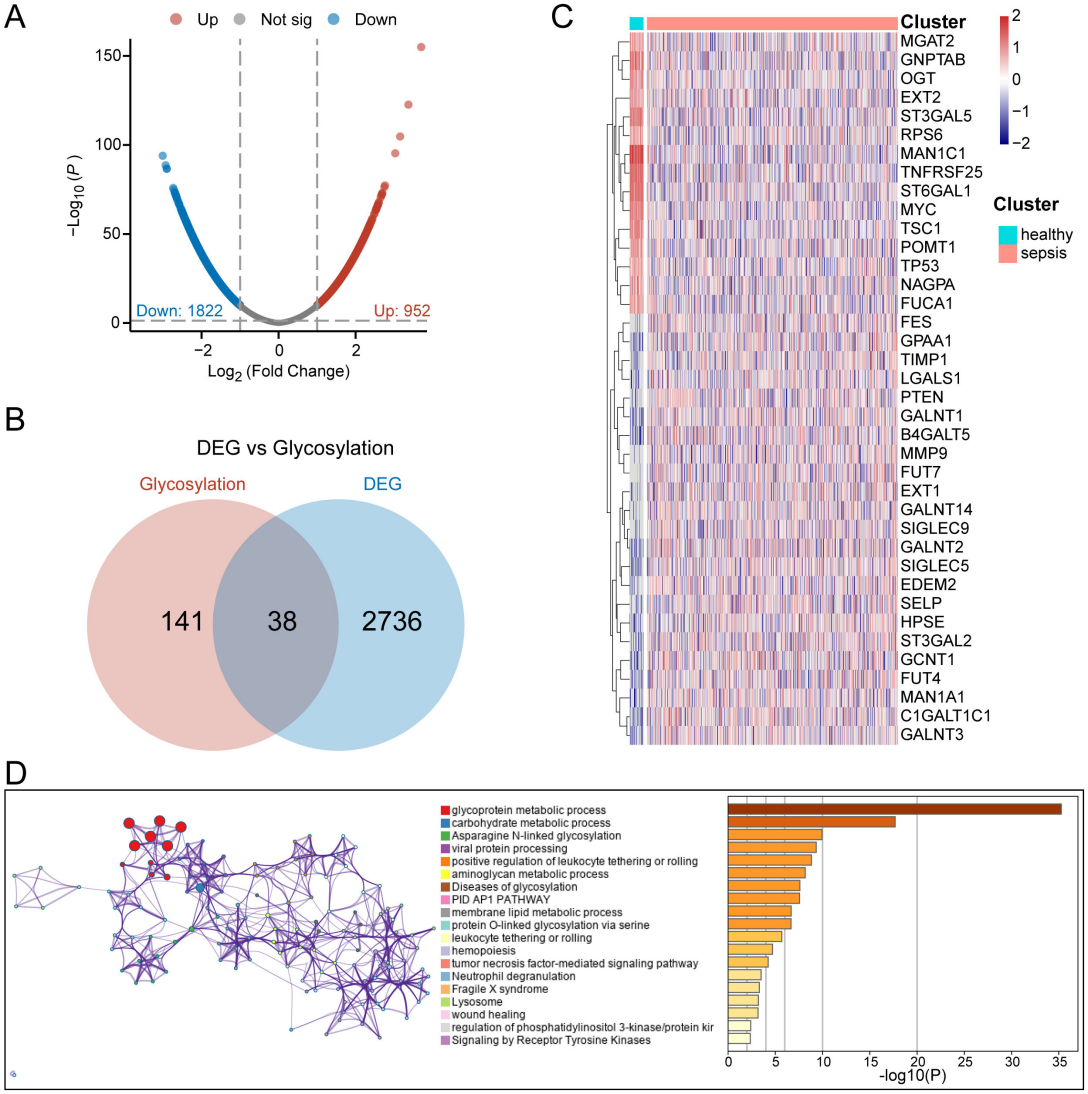
Identification of glycosylation-related differential genes. **(A)** Volcano plot of differential analysis between normal and sepsis patients. **(B)** Venn diagram for the selection of glycosylation-related differential genes. **(C)** Heatmap of gene expression for 38 glycosylation-related differential genes. **(D)** Functional enrichment diagram for 38 glycosylation-related differential genes.

The workflow of the present study is illustrated in **Figure 1**. To identify potential glycosylation-related biomarkers in sepsis, we first screened for differentially expressed genes using the “limma” R package on the GSE65682 dataset, which contains RNA-seq data from peripheral blood of sepsis patients and normal controls. This identified 2,774 differentially expressed genes, with 952 being upregulated and 1,822 being downregulated in sepsis patients (**Figure 2A**). We further screened for glycosylation-related genes based on the GGDB database, identifying 38 differentially expressed genes (**Figure 2B**). Among these, 23 were upregulated and 15 were downregulated in sepsis patients (**Figure 2C**). Functional enrichment analysis using the Metascape database revealed that these 38 genes were significantly enriched in multiple glycosylation-related pathways (**Figure 2D**).

### Identification of sepsis subtypes with different glycosylation states

**Figure 3:**
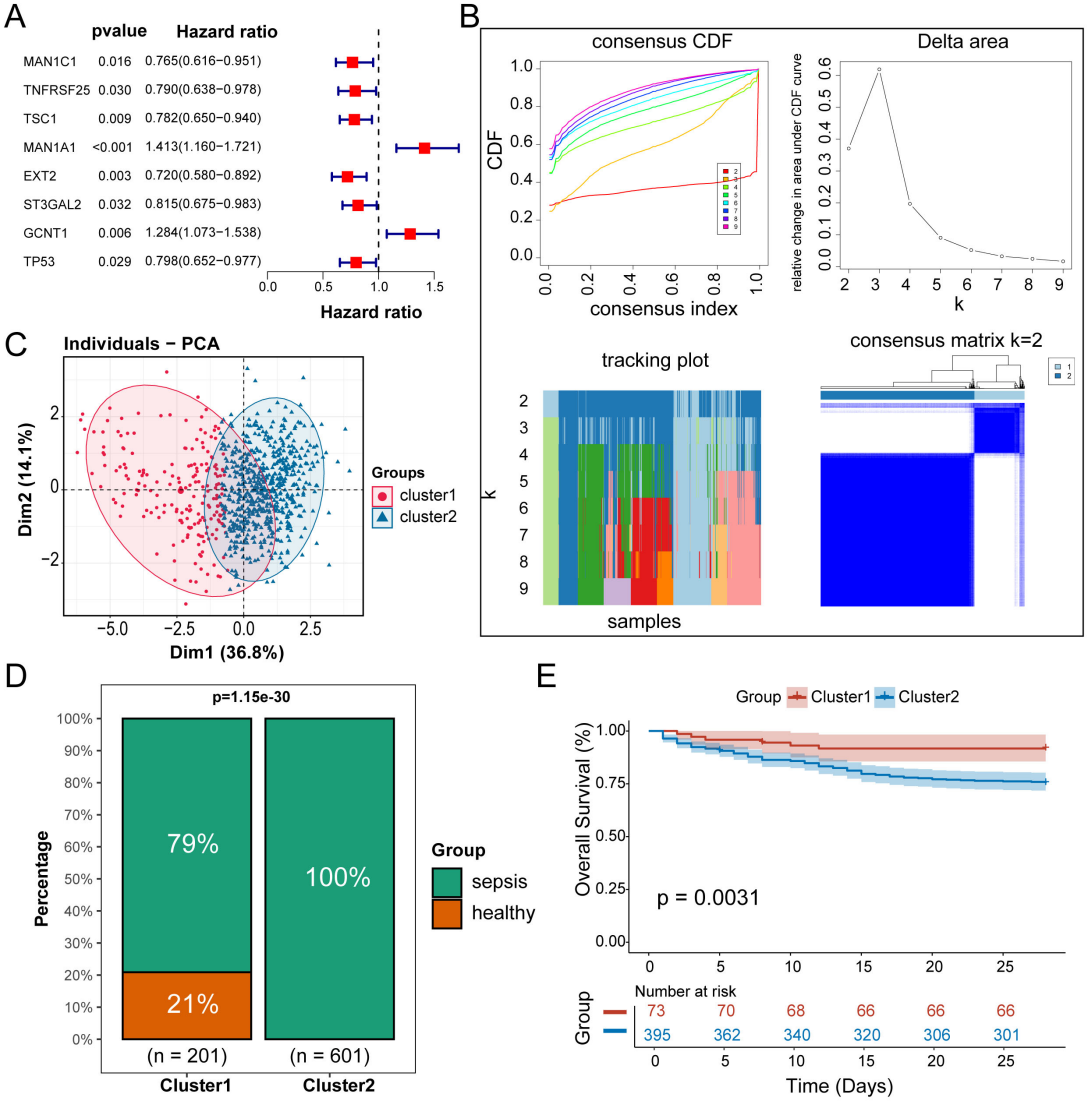
Identification of sepsis subtypes with different glycosylation states. **(A)** Forest plot of univariable Cox regression analysis. **(B)** Unsupervised clustering classifying all samples into 2 subtypes. **(C)** Principal component analysis of the samples. **(D)** Bar chart of the percentage distribution of disease status among subtypes. **(E)** KM survival curves for subtypes of sepsis patients.

To identify Hub-Gly affecting patient prognosis, we conducted a univariable Cox regression analysis, selecting 8 Hub-Glys with significant prognostic impact (**Figure 3A**). Based on these Hub-Glys, consensus clustering analysis was performed, dividing the 802 samples in GSE65682 into two subtypes (Subtype1 and Subtype2) (**Figure 3B**). PCA showed clear discrimination between these subtypes (**Figure 3C**). Disease status distribution indicated that healthy patients were concentrated in Subtype1, with a significant difference in sepsis status between the subtypes (*p*=1.15e-30) (**Figure 3D**). After excluding healthy and duplicate samples, Kaplan-Meier survival analysis was conducted on 479 sepsis patients. The survival curves demonstrated a significant difference in prognosis, with Subtype1 patients exhibiting better outcomes (**Figure 3E**).

### Establishment of prognostic models based on GRS

**Figure 4:**
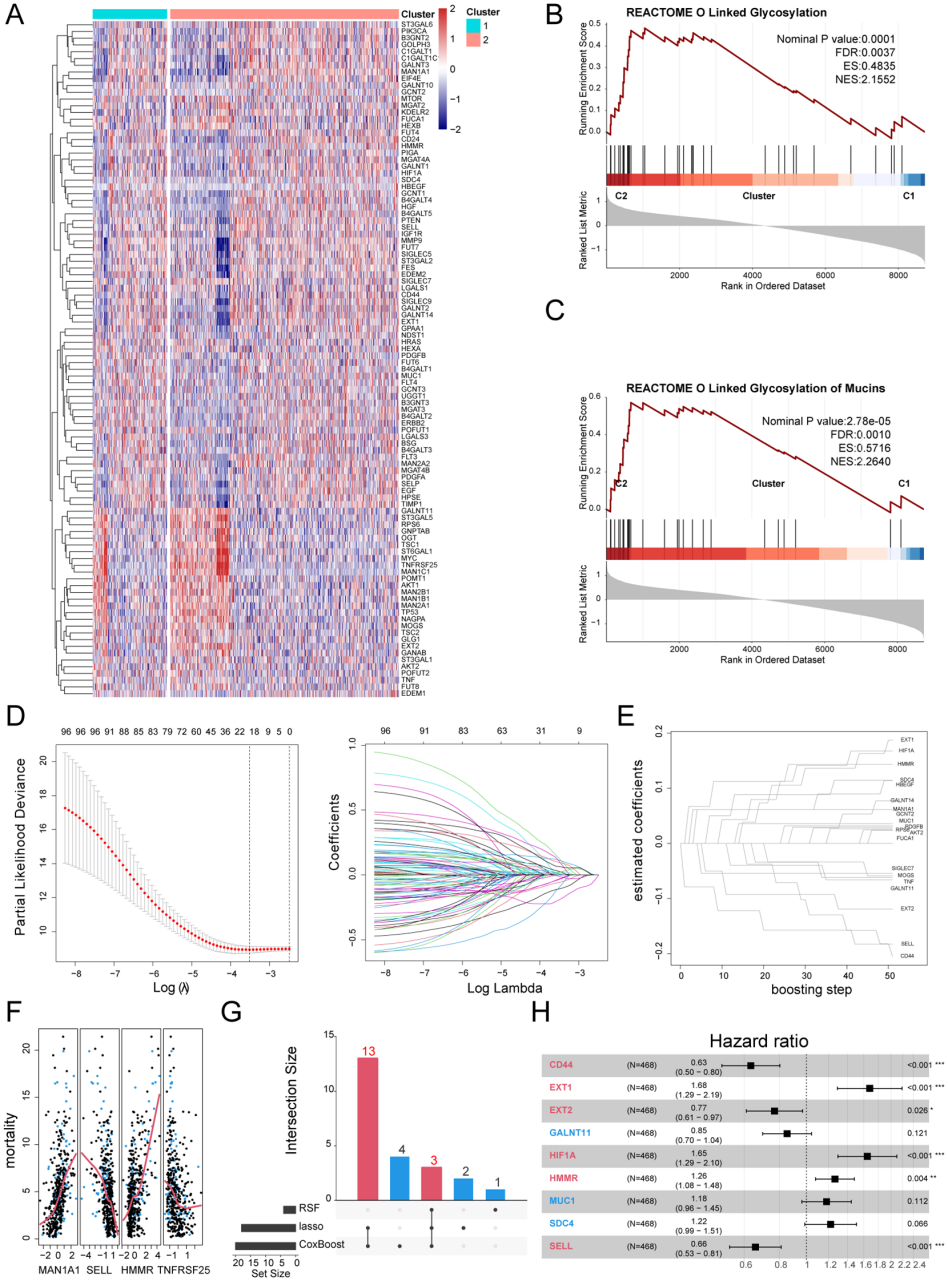
Establishment of prognostic models based on GRS. **(A)** Heatmap of Gly-DEGs between subtypes. **(B)** GSEA enrichment analysis of the O-Linked Glycosylation of Mucins pathway. **(C)** GSEA enrichment analysis of the O-Linked Glycosylation pathway. **(D)** Prognostic genes screened by the LASSO algorithm. **(E)** Prognostic genes screened by the CoxBoost algorithm. **(F)** Prognostic genes screened by the RSF algorithm. **(G)** UpSet plot of machine learning results. **(H)** Forest plot of the multivariable Cox regression analysis.

We conducted differential expression analysis between the two subtypes and identified 100 Gly-DEGs (**Figure 4A**). Reactome pathway enrichment analysis revealed that these Gly-DEGs are significantly enriched in glycosylation-related pathways, such as REACTOME O-Linked Glycosylation (**Figure 4B**) and REACTOME O-Linked Glycosylation of Mucins (**Figure 4C**).

Using the training set GSE65682, we applied multiple machine learning algorithms to select key glycosylation-related prognostic factors from these 100 Gly-DEGs. The Lasso algorithm identified 18 important prognostic factors (The full names of all gene abbreviations in this article can be found in **Supplementary Table 2**): MAN1A1, EXT2, GALNT11, CD24, EXT1, MOGS, HMMR, HIF1A, CD44, SELL, MUC1, SDC4, PDGFB, SIGLEC7, GALNT10, GCNT2, HBEGF, TNF (**Figure 4D**). The CoxBoost algorithm identified 20 important factors: MAN1A1, RPS6, EXT2, GALNT11, GALNT14, EXT1, MOGS, HMMR, FUCA1, AKT2, HIF1A, CD44, SELL, MUC1, SDC4, PDGFB, SIGLEC7, GCNT2, HBEGF, TNF (**Figure 4E**). The RSF algorithm identified 4 important factors: MAN1A1, SELL, HMMR, TNFRSF25 (**Figure 4F**). Integrating results from multiple algorithms enhances the stability of the prognostic model. Thus, we considered glycosylation-related prognostic factors selected by at least two algorithms as potential clinically relevant prognostic factors, yielding a total of 16 potential prognostic factors for sepsis (**Figure 4G**). Multivariable Cox regression analysis of these factors established the following sepsis prognosis model based on their coefficients:

GRS = CD44*(-0.46) + EXT1*10.52 + EXT2* (-0.26) + GALNT11*(-0.16) + HIF1A*0.50 + HMMR*0.23 + MUC*10.16 + SDC*40.20 + SELL*(-0.42),

where 6 factors demonstrated independent prognostic ability: CD44, EXT1, EXT2, HIF1A, HMMR, SELL (**Figure 4H**). Their functions in immune regulation or sepsis reported by previous literatures were summarized in **Supplementary Table 3**.

### Validation of GRS reliability

**Figure 5:**
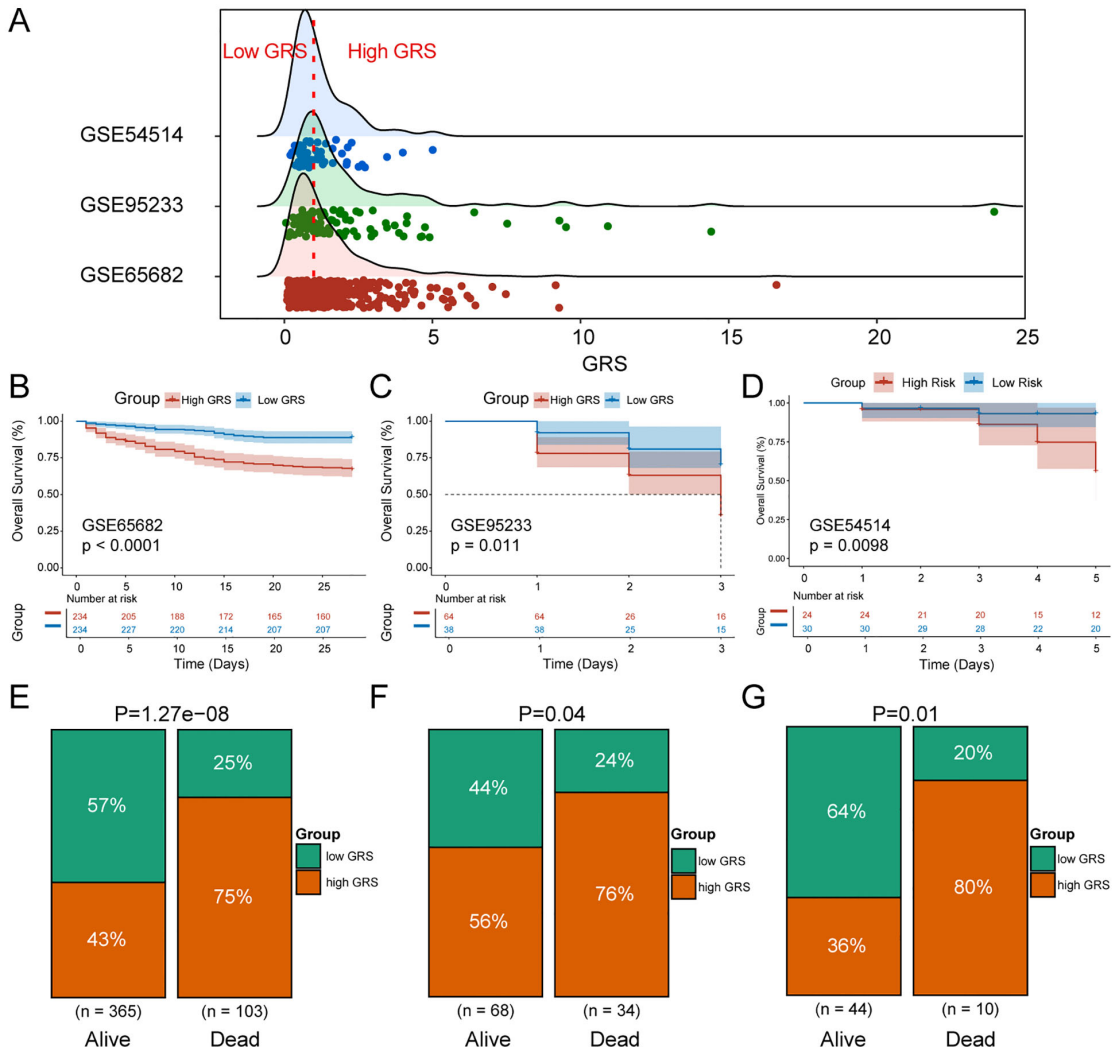
Validation of GRS reliability. **(A)** Ridge plot of GRS score distribution in the training and validation sets. **(B)** KM survival curves for high and low GRS sepsis groups in GSE65682. **(C)** KM survival curves for high and low GRS sepsis groups in GSE95233. **(D)** KM survival curves for high and low GRS sepsis groups in GSE54514. **(E)** Percentage distribution of survival states of high and low GRS groups in GSE65682. **(F)** Percentage distribution of survival states of high and low GRS groups in GSE95233. **(G)** Percentage distribution of survival states of high and low GRS groups in GSE54514.

To verify the reliability of the GRS in predicting patient prognosis, we conducted external validation using the datasets of GSE65682, GSE95233 and GSE54514. Sepsis patients were divided into high GRS and low GRS risk groups based on the median GRS value in the training set GSE65682 (**Figure 5A**). Significant survival differences were observed between the high and low GRS groups in GSE65682 (*p*<0.0001) (**Figure 5B**), GSE95233 (P=0.011) (**Figure 5C**), and GSE54514 (*p*=0.0098) (**Figure 5D**). The bar chart revealed significant distribution differences in survival states between the high and low GRS groups. In the GSE65682 dataset, High GRS group patients accounted for 75% of fatal cases, with a significant difference between groups (*p*=1.27e-08) (**Figure 5E**). In the GSE95233 dataset, High GRS group patients accounted for 76% of fatal cases, with a significant difference (*p*=0.04) (**Figure 5F**). Finally, in the GSE54514 dataset, High GRS group patients accounted for 80% of fatal cases, with a significant difference (*p*=0.01) (**Figure 5G**).

To explore the distribution of clinical characteristics among patients with different GRS states, we created a heatmap of clinical information for dataset GSE65682 (**Supplementary Figure S1A**) and dataset GSE95233 (**Supplementary Figure S1B**). The results showed that GRS was significantly associated with ICU-acquired infection (p<0.01) and gender (p<0.05). Furthermore, to determine the independent prognostic ability of GRS, we performed univariable Cox regression analysis (**Supplementary Figure S1C**) and multivariable Cox regression analysis (**Supplementary Figure S1D**) in GSE65682, incorporating GRS and clinical characteristics. The results indicated that GRS could serve as an independent prognostic factor for sepsis patients. This finding was further validated externally in GSE95233 (**Supplementary Figures S1E, F**), confirming its independent prognostic capability. To evaluate the generalizability and discriminative power of predictive factors across datasets, we calculated the C-index for each model in these three independent sepsis-related cohorts. As shown in **Supplementary Figure S3A**, C-index values varied across both datasets and predictors, reflecting differences in model robustness and cohort characteristics. GRS achieved high C-index values across multiple datasets, indicating strong and stable discriminatory performance.

### Diagnostic capability of GRS for sepsis patients

**Figure 6:**
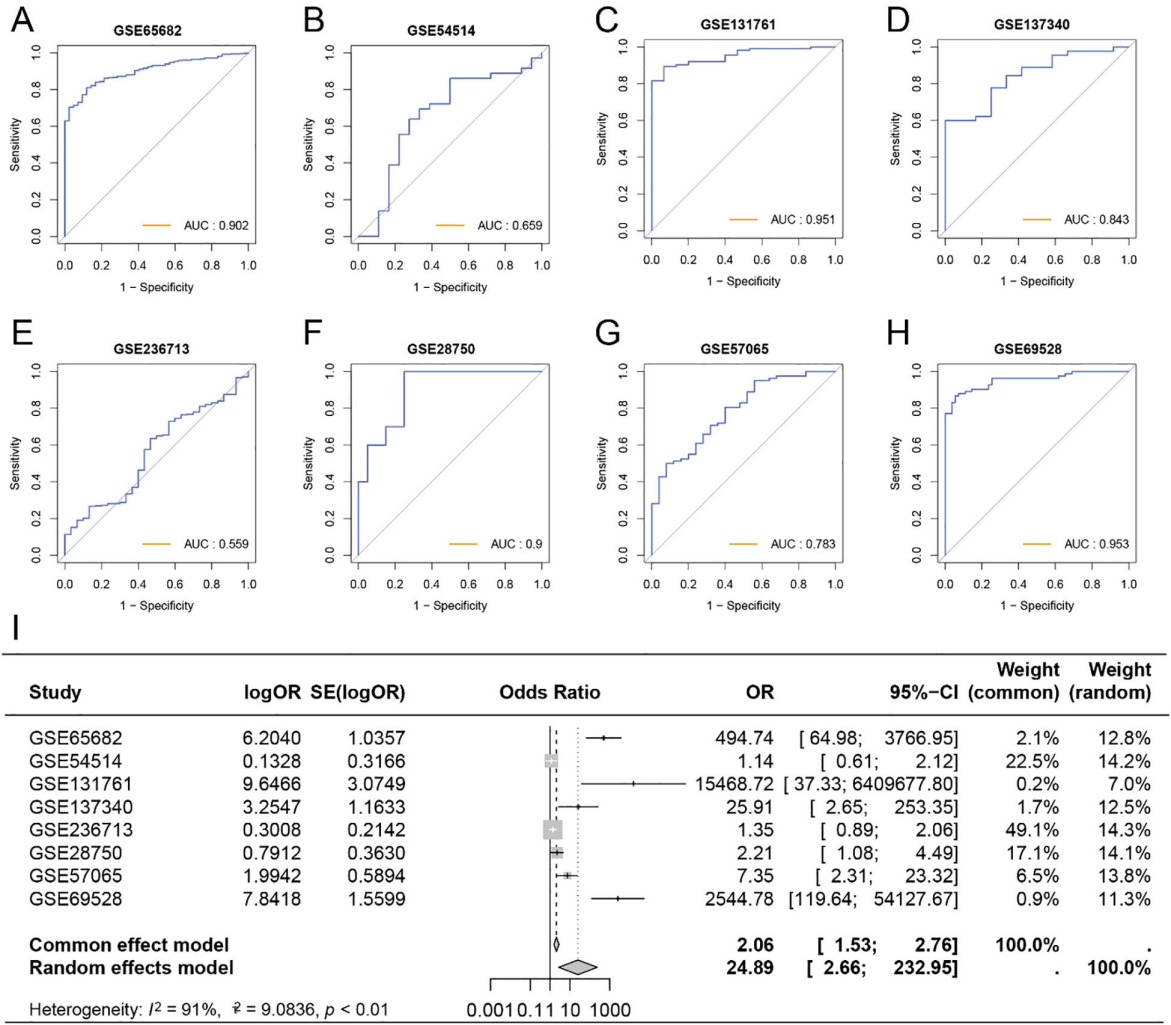
Diagnostic capability of GRS for sepsis patients. **(A)** ROC curve of GRS for diagnosing sepsis in GSE65682. **(B)** ROC curve of GRS for diagnosing sepsis in GSE54514. **(C)** ROC curve of GRS for diagnosing sepsis in GSE131761. **(D)** ROC curve of GRS for diagnosing sepsis in GSE137340. **(E)** ROC curve of GRS for diagnosing sepsis in GSE236713. **(F)** ROC curve of GRS for diagnosing sepsis in GSE28750. **(G)** ROC curve of GRS for diagnosing sepsis in GSE57065. **(H)** ROC curve of GRS for diagnosing sepsis in GSE69528. **(I)** Meta-analysis of the diagnostic efficacy of GRS for sepsis patients.

To further investigate the role of GRS in diagnosing sepsis, we generated ROC curves using GRS across multiple datasets containing both healthy individuals and sepsis patients. The results showed that the AUC values for GRS were consistently around 0.80, indicating excellent diagnostic capability (**Figures 6A–H**). This suggests that GRS is a reliable tool for diagnosing sepsis. Additionally, a meta-analysis revealed that the summary OR for GRS was 24.89 (95% *CI* = 2.66-232.95) (**Figure 6I**). We also assessed the diagnostic performance of six independent prognostic factors. **Supplementary Figures S2A-F** show that CD44, EXT1, EXT2, HIF1A, HMMR, and SELL all demonstrated good diagnostic performance.

### Glycosylation characteristics of sepsis at the single-cell level

**Figure 7:**
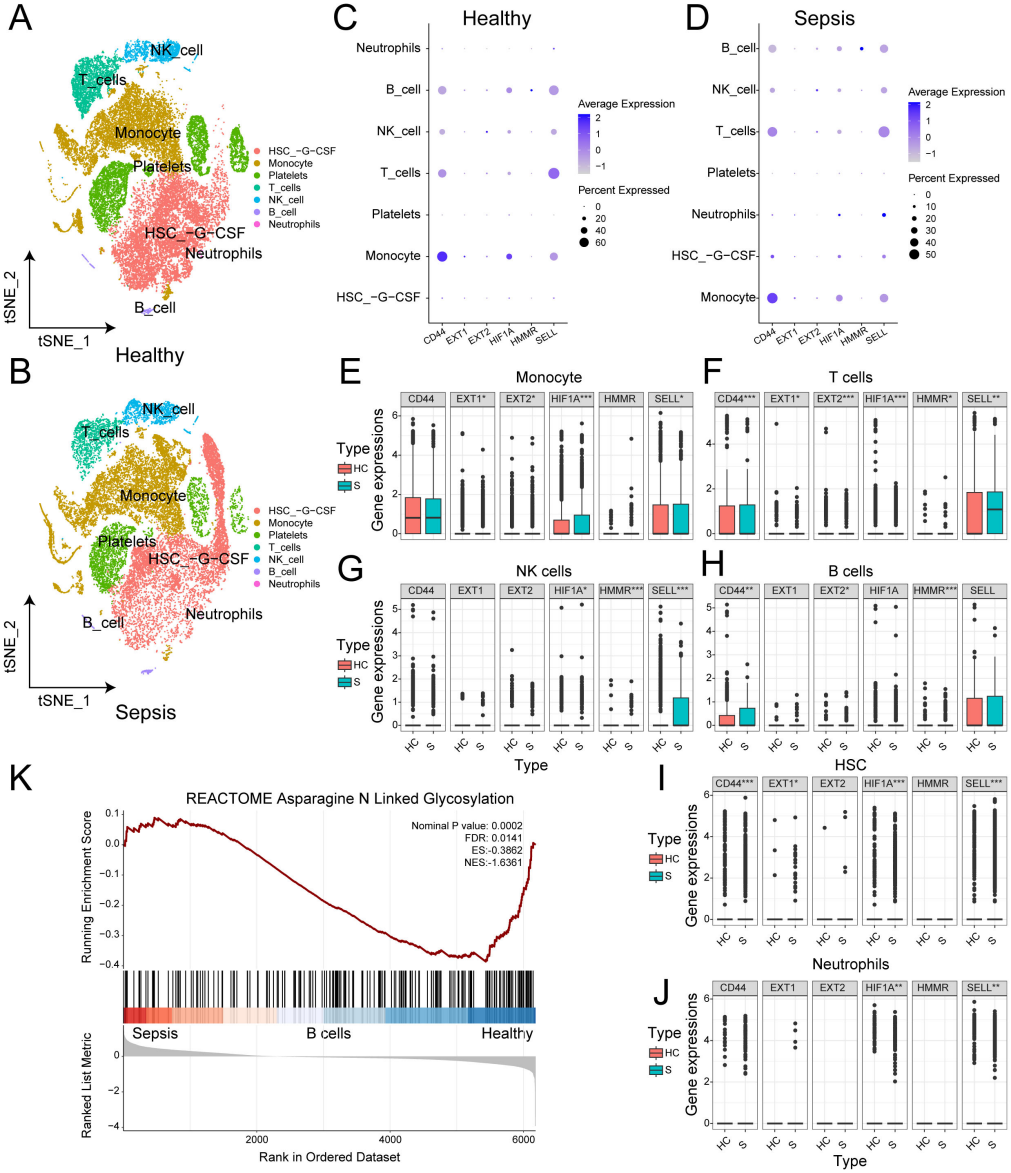
Glycosylation characteristics of sepsis at the single-cell level. **(A)** t-SNE plot of the distribution of various cell types in normal samples. **(B)** t-SNE plot of the distribution of various cell types in sepsis samples. **(C)** Bubble chart of the expression of 6 genes in different cell types in normal samples. **(D)** Bubble chart of the expression of 6 genes in different cell types in sepsis samples. **(E)** Box plot of the expression differences of 6 genes in monocytes between normal and sepsis samples. **(F)** Box plot of the expression differences of 6 genes in T cells between normal and sepsis samples. **(G)** Box plot of the expression differences of 6 genes in NK cells between normal and sepsis samples. **(H)** Box plot of the expression differences of 6 genes in B cells between normal and sepsis samples. **(I)** Box plot of the expression differences of 6 genes in HSC between normal and sepsis samples. **(J)** Box plot of the expression differences of 6 genes in neutrophils between normal and sepsis samples. **(K)** GSEA enrichment analysis between normal and sepsis samples.

To reveal glycosylation differences between sepsis patients and healthy individuals at the single-cell level, we analyzed 21,644 cell samples from four sepsis patients and 27,808 cell samples from five healthy controls. No obvious batch effects were observed in tSNE plot of different samples (**Supplementary Figure S3B**). We identified seven cell types in both normal and sepsis samples: hematopoietic stem cells (HSCs), monocytes, platelets, T cells, natural killer (NK) cells, B cells, and neutrophils (**Figures 7A, B**). Cell-type–specific differential gene expression analysis validated the annotation results (**Supplementary Table 4**).

We assessed the expression of six independent prognostic factors in these cell types and found varying levels of expression in all cell types except platelets (**Figures 7C, D**). Differential expression analysis showed that, except for CD44, the other five genes exhibited significant differences in monocytes (**Figure 7E**). All six genes showed significant differences in T cells (**Figure 7F**). In NK cells, HIF1A, HMMR, and SELL showed significant differences (**Figure 7G**). In B cells, CD44, EXT2, and HMMR were significantly different (**Figure 7H**). In HSCs, all genes except EXT2 and HMMR showed significant differences (**Figure 7I**). In neutrophils, HIF1A and SELL showed significant differences (**Figure 7J**). The details of Wilcoxon rank-sum test results were presented on **Supplementary Table 5**. To validate our findings, we applied the same analysis in GSE167363 (**Supplementary Figures S3C, D**, **S4**). Significant differences were also observed. Overall, the six independent prognostic factors showed significant differential expression in most major cell types, which may underlie their strong diagnostic capability. GSEA enrichment analysis revealed significant differences in the N-linked glycosylation pathway between normal and sepsis samples (**Figure 7K**).

**Figure 8:**
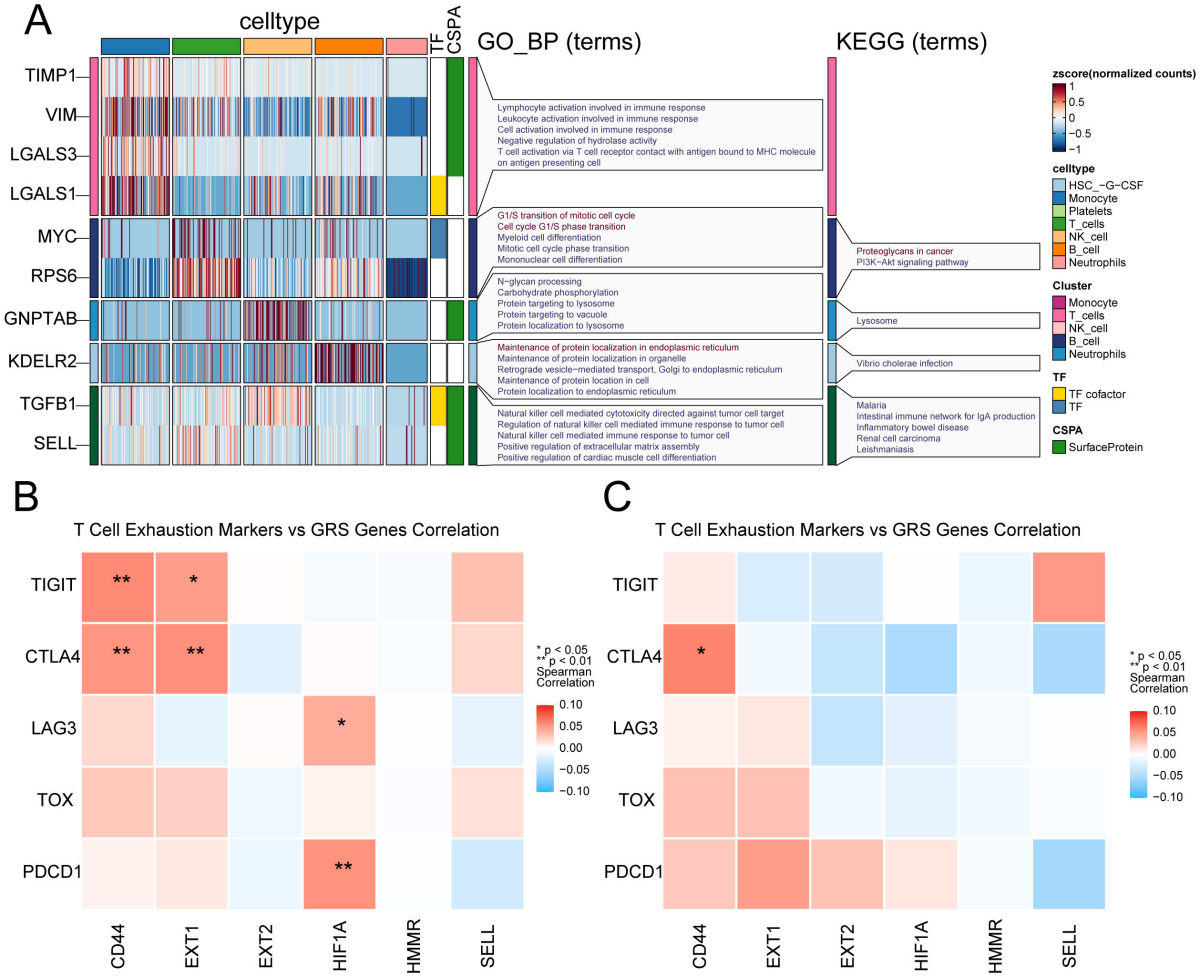
Cell-type-specific glycosylation patterns in sepsis immune cells. **(A)** Glycosylation-related genes show distinct expression and functional enrichment patterns across immune cell types in sepsis. **(B)** Heatmap shows the correlation between six GRS genes and T cell exhausting markers in healthy donors. **(C)** Heatmap shows the correlation between six GRS genes and T cell exhausting markers in sepsis patients. * indicates p < 0.05, ** indicates p < 0.01.

Aiming at the glycosylation-related immune dysfunction, we visualized the differentially expressed glycosylation-related genes and performed enrichment analysis among immune cell types (**Figure 8A**). Monocytes exhibited high expression of TIMP1, VIM, LGALS3, and LGALS1, enriched in pathways related to immune activation and antigen presentation, indicating glycosylation’s role in monocyte-driven inflammatory responses. T cells upregulated MYC and RPS6, enriched in cell cycle and lysosomal pathways, suggesting glycosylation’s involvement in T cell proliferation and exhaustion priming. NK cells showed elevated expression of GNPTAB and KDELR2, with enrichment in N-glycan processing and pathogen response pathways, linking glycosylation to cytotoxic granule trafficking and effector function. B cells demonstrated ER-related enrichment in protein folding and retrograde transport, implicating glycosylation in antibody maturation. These findings reveal cell-type-specific glycosylation signatures that contribute to immune dysfunction in sepsis. Genes such as MYC (a transcription factor, TF), SELL, and LGALS3 (associated with cold shock domain-containing protein A) may act as upstream regulators or potential therapeutic targets in the context of glycosylation dysfunction related to sepsis. Then, we performed correlation analysis between six GRS genes and exhaustion markers in T cells (**Figures 8B, C**). We observed stronger correlations between glycosylation-related genes and T cell exhaustion markers in normal T cells compared to sepsis-derived T cells. This suggests that glycosylation may normally act in coordination with exhaustion-related transcriptional programs, but this relationship is disrupted under septic conditions, possibly contributing to dysfunctional immune responses.

### Expression validation of key glycosylation-related genes in patient samples

**Figure 9:**
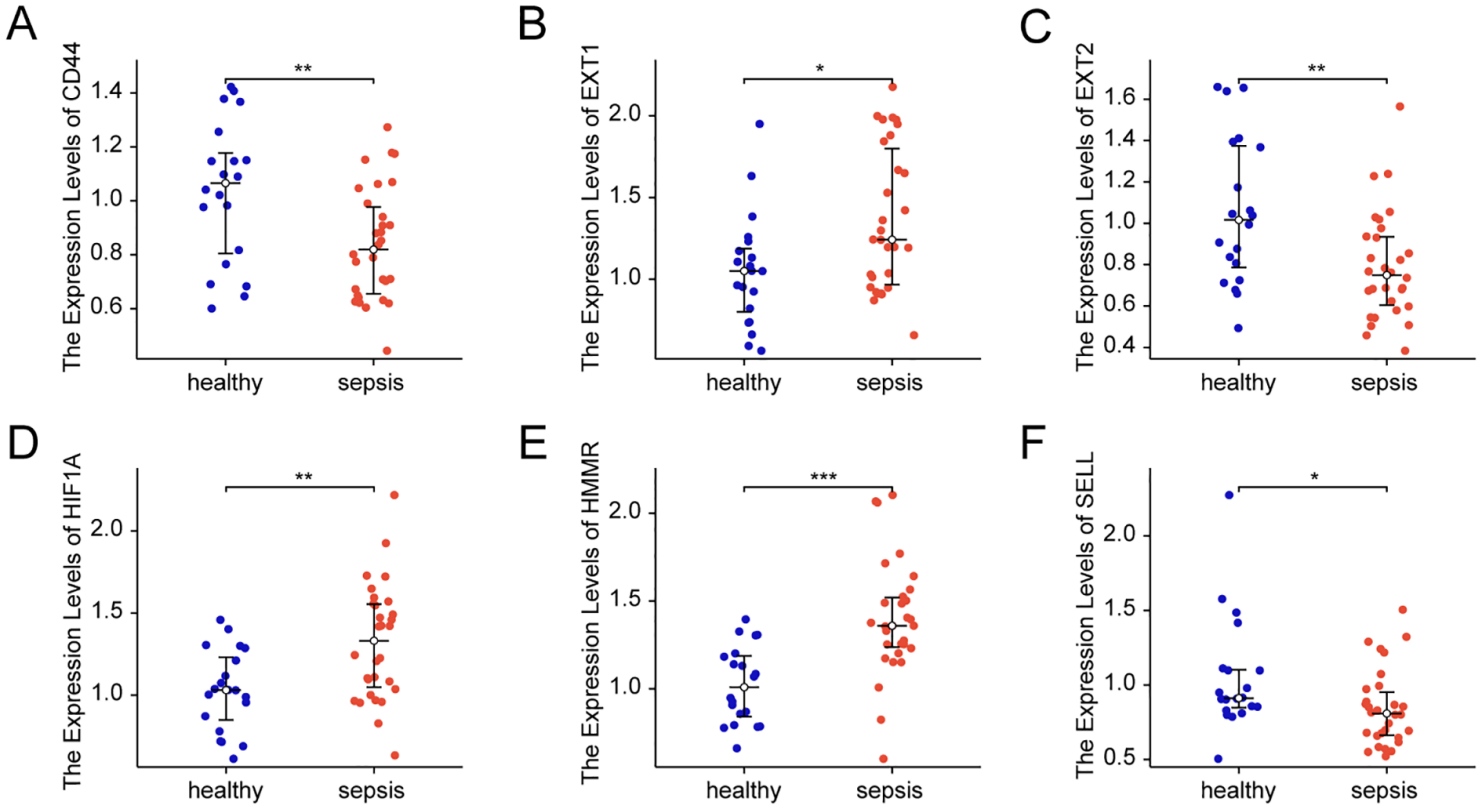
Expression of key glycosylation-related genes in patient samples. **(A)** mRNA Expression value of CD44 in patient samples. **(B)** mRNA Expression value of EXT1 in patient samples. **(C)** mRNA Expression value of EXT2 in patient samples. **(D)** mRNA Expression value of HIF1A in patient samples. **(E)** mRNA Expression value of HMMR in patient samples. **(F)** mRNA Expression value of SELL in patient samples. All data are presented as median ± interquartile range (IQR) and were analyzed using the Wilcoxon rank-sum test. * indicates p < 0.05, ** indicates p < 0.01 and *** indicates p < 0.001.

We enrolled 30 sepsis patients, with their clinical information listed in **Supplementary Table 6**. We validated the expression of key glycosylation-related genes in peripheral blood cells through PCR. The results showed that, compared with the healthy control group, the expression of EXT1, HIF1A and HMMR was upregulated in sepsis, whereas the expression of CD44, EXT2 and SELL was downregulated in sepsis group (**Figures 9A–F**).

## Discussion

In recent years, the role of glycosylation in sepsis has garnered increasing attention. However, there has been no systematic study on glycosylation genes in sepsis to date. This study identified differentially expressed glycosylation genes associated with sepsis through bioinformatics analysis. Using consensus clustering methods, patients were classified into different subtypes, and survival analysis was employed to assess the prognostic value of these subtypes. Furthermore, machine learning techniques such as random forest, CoxBoost algorithm, and LASSO regression were used to identify key genes related to patient prognosis and to establish a GRS model. The reliability of the GRS model was validated using external datasets, and its diagnostic and prognostic value was examined through meta-analysis. Additionally, the study evaluated the relationship between GRS and clinical characteristics and analyzed the expression differences of GRS model genes at the single-cell level. Ultimately, our findings suggest that GRS is a promising tool for predicting the prognosis of sepsis.

Sepsis exhibits a high degree of heterogeneity, making its diagnosis and treatment challenging (6). To better understand and address this complexity, researchers have postulated the concept of sepsis subtypes (25). These subtypes can be classified based on various biological characteristics, including genotyping and immune status (25, 26). This study focuses on the analysis of glycosylation subtypes, a relatively new research perspective. Through our research, we discovered that specific glycosylation-related gene expression patterns are significantly associated with the prognosis of sepsis patients. This suggests that glycosylation subtypes may be a valuable classification tool for sepsis, aiding in identifying patient groups with different clinical presentations and prognoses. Additionally, these findings imply that interventions targeting specific glycosylation pathways may provide new strategies for sepsis treatment.

Enrichment analysis of differentially expressed genes between the two subtypes revealed significant enrichment in the O-linked glycosylation and O-linked glycosylation of mucins pathways. O-linked glycosylation is a post-translational modification process that involves adding sugar molecules to the serine or threonine residues of proteins (27). In sepsis, proper glycosylation helps to regulate immune responses and facilitates intercellular communication and signal transduction (28). Mucins are highly glycosylated proteins widely present on the surface of epithelial cells and in mucous secretions, and their O-glycosylation plays a critical role in maintaining epithelial barrier function (29). Although the protective role of O-glycosylation in sepsis is significant, the O-glycosylation of certain proteins can have negative effects (30, 31). For example, a study indicated that the O-glycosylation pattern of some proteins in sepsis patients change, resulting in excessive O-glycosylation (30). Endoplasmic reticulum stress and the activation of the unfolded protein response are associated with excessive O-glycosylation, leading to cellular dysfunction and organ damage (32, 33). Additionally, mucin-type O-glycans can shift the balance towards increased susceptibility to microbial infections and subsequent tissue damage (34). These findings suggest that clinical treatment should balance the regulation of O-glycosylation to maximize its protective effects while avoiding the adverse consequences of excessive glycosylation (31).

In this study, we successfully developed a GRS by integrating multiple machine learning algorithms. The random forest algorithm excelled in handling high-dimensional data and identifying important features, aiding in the selection of key genes associated with mortality (21). The CoxBoost algorithm, designed specifically for survival data, was effective in identifying genes closely related to patient prognosis (22). LASSO regression improved the model’s stability and interpretability through penalization (23). By combining the results from these algorithms, we gained a more comprehensive understanding of the molecular mechanisms of sepsis and constructed a robust prognostic model. The key genes identified, such as MAN1A1, EXT1, EXT2, HIF1A, HMMR, and SELL, demonstrated independent prognostic capabilities in multivariable Cox regression analysis. Some of these genes have been previously reported to play critical roles in the pathogenesis of sepsis. For instance, HIF1A is crucial in inflammation and angiogenesis, with changes in its expression levels potentially linked to the severity and prognosis of sepsis (35). SELL is a cell adhesion molecule involved in the rolling and migration of immune cells, and its abnormal expression may affect immune cell responses to infection (36). The identification of these key genes provides new insights for future research. Further studies could explore the specific mechanisms by which these genes contribute to sepsis and how they interact with other biological processes to influence sepsis prognosis.

Further single-cell analysis provided in-depth insights into the expression differences of GRS model genes across various cell types. We observed significant expression differences of multiple independent prognostic factors in monocytes and T cells, which may reflect the crucial role of glycosylation in regulating the immune behavior and signal transduction of these cells. Additionally, GSEA enrichment analysis revealed significant differences in the N-linked glycosylation pathway between normal and sepsis patient samples. N-linked glycosylation is a post-translational modification that occurs on asparagine residues (37). Research has shown that N-linked glycosylation plays a key role in the immune system by modifying receptors on the surface of immune cells and secreted cytokines, thus regulating immune responses (38). In sepsis patients, changes in the IgG N-linked glycosylation patterns in serum are significantly associated with immune responses and patient prognosis, further underscoring the importance of N-linked glycosylation in sepsis (39). Additionally, glycosylation remodeling is not merely a downstream response, but may serve as an active regulatory node shaping immune cell states during sepsis. This finding provides new perspectives for further mechanistic studies into glycosylation-TF-exhaustion networks and suggests potential therapeutic strategies targeting glycosylation pathways to restore immune balance in sepsis.

Despite the positive results achieved in developing and validating the GRS model, several limitations and directions for future research remain. Although the model demonstrated robust prognostic performance across multiple transcriptomic datasets, the clinical sample size used for validation was relatively small, limiting the generalizability of the findings. This constraint is primarily due to the challenges of obtaining well-characterized patient samples in the acute phase of sepsis under ethical and logistical constraints. Future studies with larger and more diverse patient cohorts are needed to confirm the model’s applicability across different clinical settings and populations. Additionally, the potential clinical utility of the GRS model—such as its use in ICU risk stratification, early triage, or glycosylation-targeted biomarker development—warrants further investigation. Integrating the GRS with other clinical parameters, including inflammatory markers, genetic background, and treatment response, may further enhance its prognostic accuracy. Moreover, in-depth exploration of the biological functions and regulatory mechanisms of key genes in the GRS model will contribute to the development of novel therapeutic strategies to improve outcomes for sepsis patients. Furthermore, whether glycosylation changes are drivers or consequences of immune dysfunction in sepsis remains unclear. Aberrant glycosylation may contribute to immune dysregulation by affecting receptor signaling or cell adhesion, whereas inflammatory stress may also disrupt glycosylation pathways. Our findings support a potential association, but further studies are needed to clarify causality.

In summary, this study provides a new tool for prognostic assessment in sepsis through innovative bioinformatics methods and machine learning techniques. The development and validation of the GRS model not only highlight the significant role of glycosylation in the pathogenesis of sepsis but also offer valuable information for future clinical research and treatment. With further research and validation, the GRS model has the potential to become an important component of sepsis management, helping to improve patient prognosis and quality of life.

## Data Availability

The original contributions presented in the study are included in the article/**Supplementary Material**. Further inquiries can be directed to the corresponding authors.

## Supplementary material

Supplementary Figure 1 Association of GRS with clinical characteristics. **(A)** Heatmap of the clinical characteristics of patients under different GRS states in GSE65682. **(B)** Heatmap of the clinical characteristics of patients under different GRS states in GSE95233. **(C)** Forest plot of the univariable Cox regression analysis in GSE65682. **(D)** Forest plot of the multivariable Cox regression analysis in GSE65682. **(E)** Forest plot of the univariable Cox regression analysis in GSE95233. **(F)** Forest plot of the multivariable Cox regression analysis in GSE95233.

Supplementary Figure 2 Meta-analysis of the diagnostic efficacy of 6 prognostic genes. **(A)** Meta-analysis of the diagnostic value of CD44 for sepsis patients. **(B)** Meta-analysis of the diagnostic value of EXT1 for sepsis patients. **(C)** Meta-analysis of the diagnostic value of EXT2 for sepsis patients. **(D)** Meta-analysis of the diagnostic value of HIF1A for sepsis patients. **(E)** Meta-analysis of the diagnostic value of HMMR for sepsis patients. **(F)** Meta-analysis of the diagnostic value of SELL for sepsis patients.

Supplementary Figure 3 Single cell data preprocessing. **(A)** Bar plot showing the C-index values of different predictive factors across multiple sepsis-related datasets. **(B)** Sample t-SNE plot shows no obvious batch effect in GSE175453. **(C)** Sample UMAP plot shows obvious batch effect in GSE176363. **(D)** Sample UMAP plot shows no obvious batch effect after harmony.

Supplementary Figure 4 Glycosylation characteristics validation. **(A)** UMAP plot of the distribution of various cell types in normal samples. **(B)** UMAP plot of the distribution of various cell types in sepsis samples. **(C)** Bubble chart of the expression of 6 genes in different cell types in normal samples. **(D)** Bubble chart of the expression of 6 genes in different cell types in sepsis samples. **(E)** Box plot of the expression differences of 6 genes in monocytes between normal and sepsis samples. **(F)** Box plot of the expression differences of 6 genes in T cells between normal and sepsis samples. **(G)** Box plot of the expression differences of 6 genes in NK cells between normal and sepsis samples. **(H)** Box plot of the expression differences of 6 genes in B cells between normal and sepsis samples. **(I)** Box plot of the expression differences of 6 genes in HSC between normal and sepsis samples. **(J)** Box plot of the expression differences of 6 genes in neutrophils between normal and sepsis samples. **(K)** GSEA enrichment analysis between normal and sepsis samples.
