# Supplementary figures and images for: Identification and validation of glycosylation-related gene signatures for prognostic stratification in sepsis
Source: Front Immunol. 2025 Jul 2;16:1608082. doi: 10.3389/fimmu.2025.1608082 (PMC12263689; doi:10.3389/fimmu.2025.1608082)

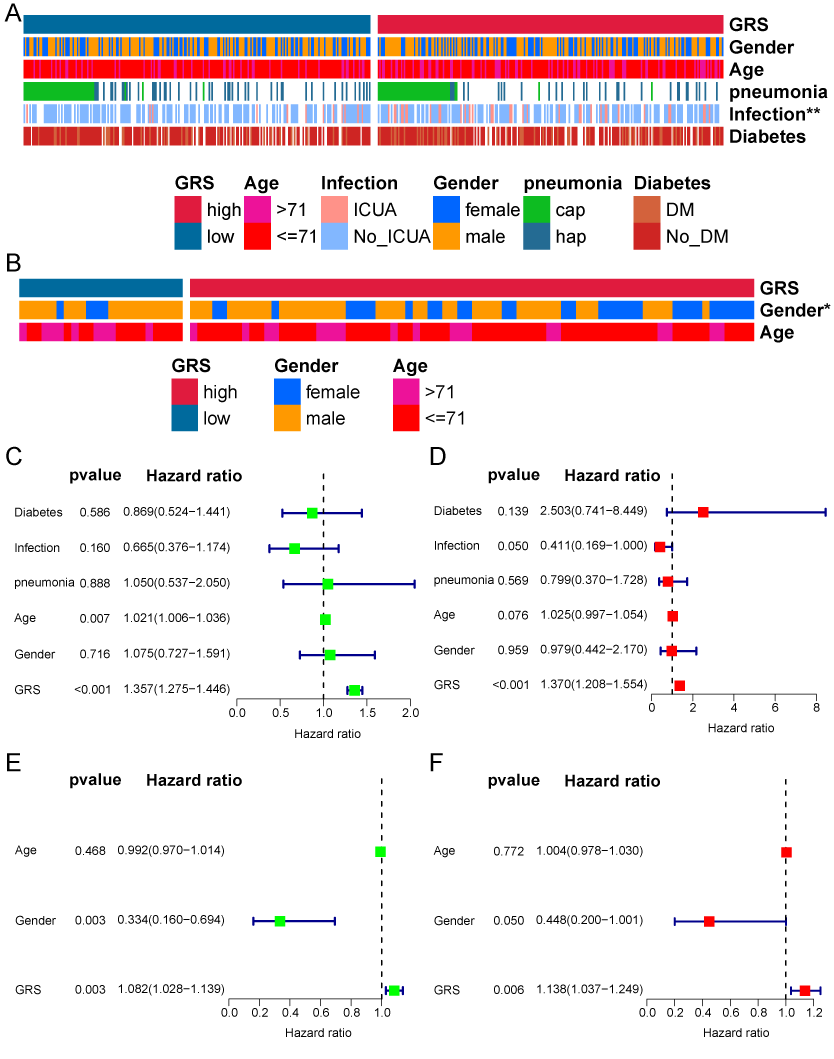

Supplement: Supplementary Figure 1 — Association of GRS with clinical characteristics. (A) Heatmap of the clinical characteristics of patients under different GRS states in GSE65682. (B) Heatmap of the clinical characteristics of patients under different GRS states in GSE95233. (C) Forest plot of the univariable Cox regression analysis in GSE65682. (D) Forest plot of the multivariable Cox regression analysis in GSE65682. (E) Forest plot of the univariable Cox regression analysis in GSE95233. (F) Forest plot of the multivariable Cox regression analysis in GSE95233. [file Image1.tif]

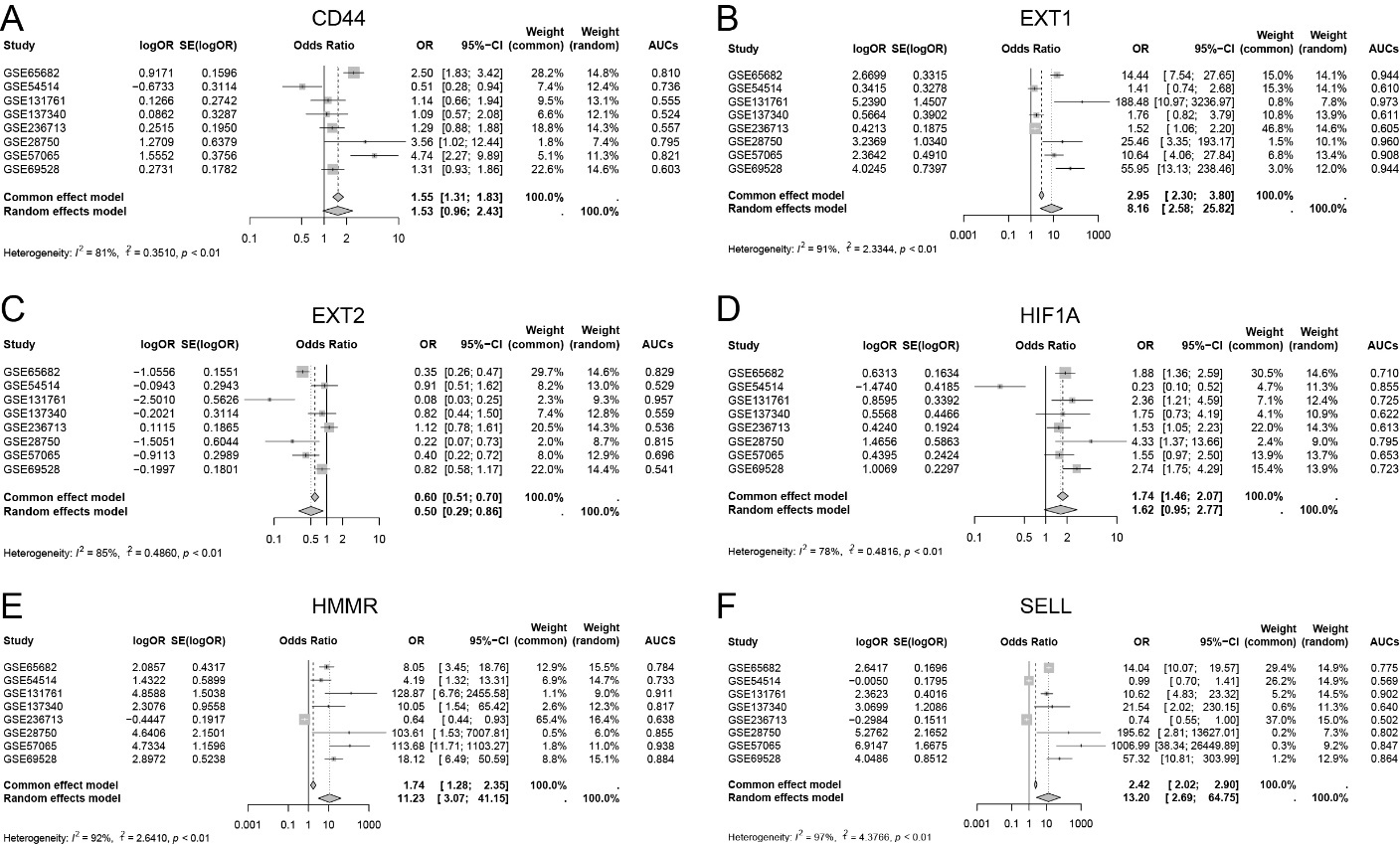

Supplement: Supplementary Figure 2 — Meta-analysis of the diagnostic efficacy of 6 prognostic genes. (A) Meta-analysis of the diagnostic value of CD44 for sepsis patients. (B) Meta-analysis of the diagnostic value of EXT1 for sepsis patients. (C) Meta-analysis of the diagnostic value of EXT2 for sepsis patients. (D) Meta-analysis of the diagnostic value of HIF1A for sepsis patients. (E) Meta-analysis of the diagnostic value of HMMR for sepsis patients. (F) Meta-analysis of the diagnostic value of SELL for sepsis patients. [file Image2.tif]

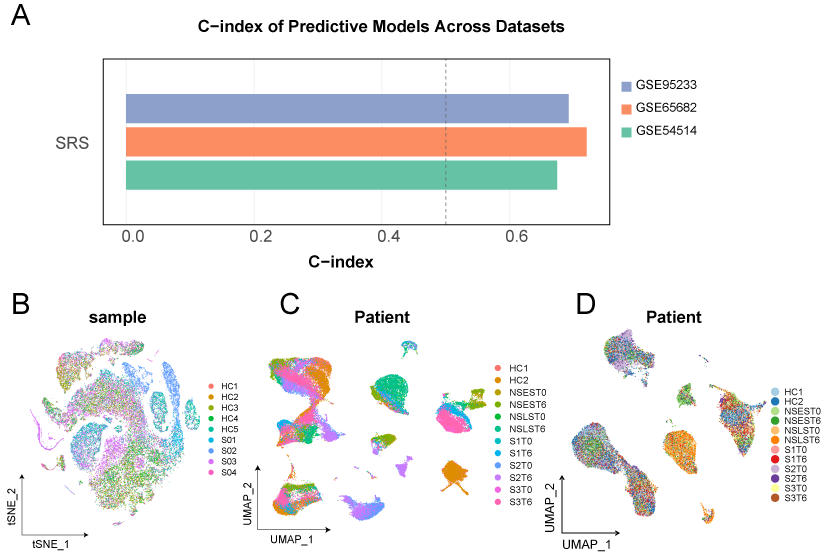

Supplement: Supplementary Figure 3 — Single cell data preprocessing. (A) Bar plot showing the C-index values of different predictive factors across multiple sepsis-related datasets. (B) Sample t-SNE plot shows no obvious batch effect in GSE175453. (C) Sample UMAP plot shows obvious batch effect in GSE176363. (D) Sample UMAP plot shows no obvious batch effect after harmony. [file Image3.tif]

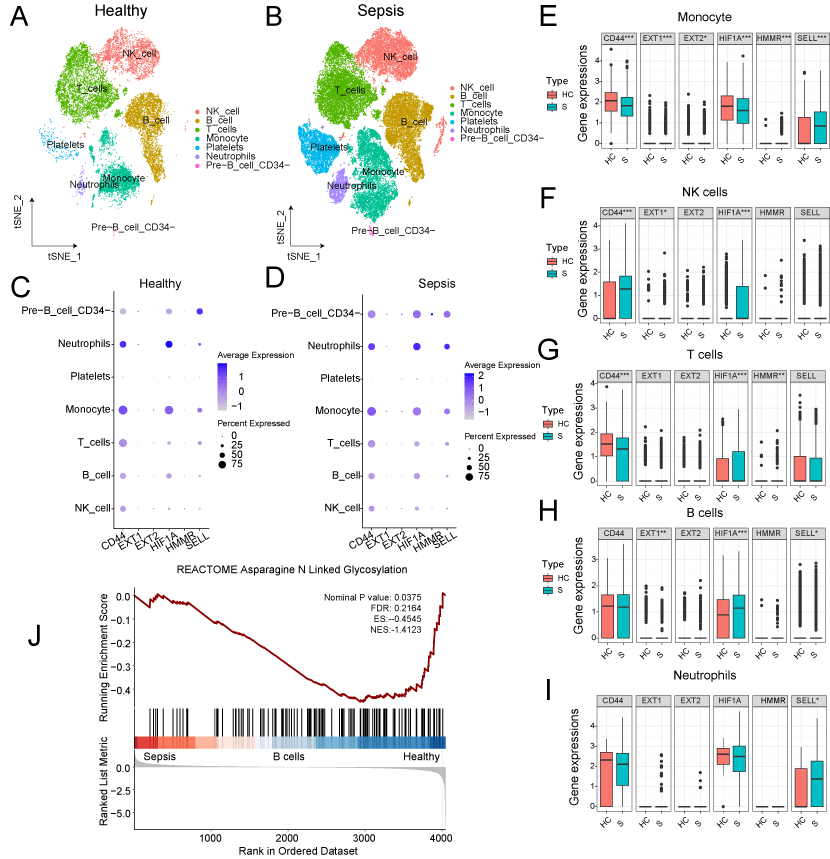

Supplement: Supplementary Figure 4 — Glycosylation characteristics validation. (A) UMAP plot of the distribution of various cell types in normal samples. (B) UMAP plot of the distribution of various cell types in sepsis samples. (C) Bubble chart of the expression of 6 genes in different cell types in normal samples. (D) Bubble chart of the expression of 6 genes in different cell types in sepsis samples. (E) Box plot of the expression differences of 6 genes in monocytes between normal and sepsis samples. (F) Box plot of the expression differences of 6 genes in T cells between normal and sepsis samples. (G) Box plot of the expression differences of 6 genes in NK cells between normal and sepsis samples. (H) Box plot of the expression differences of 6 genes in B cells between normal and sepsis samples. (I) Box plot of the expression differences of 6 genes in HSC between normal and sepsis samples. (J) Box plot of the expression differences of 6 genes in neutrophils between normal and sepsis samples. (K) GSEA enrichment analysis between normal and sepsis samples. [file Image4.tif]
