# Supplementary material for: Identification and validation of glycosylation-related gene signatures for prognostic stratification in sepsis
Source: Front Immunol. 2025 Jul 2;16:1608082. doi: 10.3389/fimmu.2025.1608082 (PMC12263689; doi:10.3389/fimmu.2025.1608082)
Supplement: Supplementary file 5 [file Table1.docx]

Supplementary Table 1. Primers of the genes in this study.

| GAPHD | F | GAAGGTGAAGGTCGGAGTC |
| --- | --- | --- |
|  | R | GGAAGATGGTGATGGGATTT |
| CD44 | F | 5´-CAAGCCACTCCAGGACAAGG-3´ |
|  | R | 5´- ATCCAAGTGAGGGACTACAACAG-3´ |
| EXT1 | F | TGTGCCTGTCGTCGTCATTGAAG |
|  | R | ACGGCGTCTGTGATGATGTTGTC |
| EXT2 | F | TGCCAGCCGACAGTCCCATC |
|  | R | GGGTTGAAGCCACAGCGATAGAC |
| HMMR | F | CCAACTCAAGCAACAGGAGGAAG |
|  | R | GAGACGCCACTTGTTAATTTCTTCAG |
| HIF1A | F | GTTCCGCAAGCCCTGAAAGC |
|  | R | TCATCAGTGGTGGCAGTGGTAG |
| SELL | F | ACAAGGAGGACTGCGTGGAGATC |
|  | R | TTAGTTTGTGGCAGGCGTCATCG |
