# Supplementary material for: Identification and validation of glycosylation-related gene signatures for prognostic stratification in sepsis
Source: Front Immunol. 2025 Jul 2;16:1608082. doi: 10.3389/fimmu.2025.1608082 (PMC12263689; doi:10.3389/fimmu.2025.1608082)
Supplement: Supplementary file 6 [file Table2.docx]

Supplementary Table 2. The full names of all gene abbreviations.

| Gene | Full name |
| --- | --- |
| AKT2 | AKT serine/threonine kinase 2 |
| EXT1 | exostosin glycosyltransferase 1 |
| EXT2 | exostosin glycosyltransferase 2 |
| FUCA1 | alpha-L-fucosidase 1 |
| GALNT10 | polypeptide N-acetylgalactosaminyltransferase 10 |
| GALNT11 | polypeptide N-acetylgalactosaminyltransferase 11 |
| GALNT14 | polypeptide N-acetylgalactosaminyltransferase 14 |
| GCNT2 | glucosaminyl (N-acetyl) transferase 2 |
| HBEGF | heparin binding EGF like growth factor |
| HIF1A | hypoxia inducible factor 1 subunit alpha |
| HMMR | hyaluronan mediated motility receptor |
| MAN1A1 | mannosidase alpha class 1A member 1 |
| MOGS | mannosyl-oligosaccharide glucosidase |
| MUC1 | mucin 1 |
| PDGFB | platelet derived growth factor subunit B |
| RPS6 | ribosomal protein S6 |
| SDC4 | syndecan 4 |
| SELL | selectin L |
| SIGLEC7 | sialic acid binding Ig like lectin 7 |
| TNF | tumor necrosis factor |
| TNFRSF25 | TNF receptor superfamily member 25 |
