# Supplementary material for: Identification and validation of glycosylation-related gene signatures for prognostic stratification in sepsis
Source: Front Immunol. 2025 Jul 2;16:1608082. doi: 10.3389/fimmu.2025.1608082 (PMC12263689; doi:10.3389/fimmu.2025.1608082)
Supplement: Supplementary file 7 [file Table3.docx]

Supplementary Table 3.The literature evidence summarizing for each gene’s function in immune regulation or sepsis.

| Gene | Full Name | Role in Immune Regulation / Sepsis | Key References |
| --- | --- | --- | --- |
| CD44 | Cluster of Differentiation 44 | Mediates leukocyte adhesion, migration, and cytokine signaling in inflammation and infection | Buscher et al., Nat Commun, 2016; Heindel et al., ACS Infect Dis, 2022 |
| EXT1 | Exostosin Glycosyltransferase 1 | Involved in heparan sulfate biosynthesis; may influence immune cell signaling via ECM | Loke et al., Mol Aspects Med, 2016 |
| EXT2 | Exostosin Glycosyltransferase 2 | Forms complex with EXT1; modulates glycan structure impacting cell communication | Schjoldager et al., Nat Rev Mol Cell Biol, 2020 |
| HIF1A | Hypoxia-Inducible Factor 1-alpha | Regulates inflammatory gene expression under hypoxia; elevated in septic leukocytes | Ferreira et al., Shock, 2021 |
| HMMR | Hyaluronan Mediated Motility Receptor | Promotes leukocyte motility and cytoskeleton remodeling in inflammation | Wu et al., Redox Biol, 2023 |
| SELL | Selectin L (L-selectin) | Mediates leukocyte rolling and extravasation into inflamed tissue | Buscher et al., Nat Commun, 2016 |
