# Supplementary material for: Identification and validation of glycosylation-related gene signatures for prognostic stratification in sepsis
Source: Front Immunol. 2025 Jul 2;16:1608082. doi: 10.3389/fimmu.2025.1608082 (PMC12263689; doi:10.3389/fimmu.2025.1608082)
Supplement: Supplementary file 9 [file Table5.docx]

Supplementary Table 5. Summarizing of the mean, standard deviation, and p-value for each gene in each cell type across both groups.

| **Cell Type** | **Gene** | **Group** | **Mean** | **SD** | **n Cells** | **p-value** |
| --- | --- | --- | --- | --- | --- | --- |
| Monocyte | CD44 | Sepsis | 0.937 | 1.01 | 7763 | 0.22698 |
|  | CD44 | Control | 0.963 | 1.051 | 10487 | 0.22698 |
|  | EXT1 | Sepsis | 0.052 | 0.263 | 7763 | 0.0305 |
|  | EXT1 | Control | 0.044 | 0.246 | 10487 | 0.0305 |
|  | EXT2 | Sepsis | 0.03 | 0.21 | 7763 | 0.02037 |
|  | EXT2 | Control | 0.034 | 0.21 | 10487 | 0.02037 |
|  | HIF1A | Sepsis | 0.471 | 0.791 | 7763 | 0 |
|  | HIF1A | Control | 0.396 | 0.739 | 10487 | 0 |
|  | HMMR | Sepsis | 0.003 | 0.083 | 7763 | 0.05422 |
|  | HMMR | Control | 0.001 | 0.032 | 10487 | 0.05422 |
|  | SELL | Sepsis | 0.719 | 0.959 | 7763 | 0.01781 |
|  | SELL | Control | 0.692 | 0.968 | 10487 | 0.01781 |
| T cells | CD44 | Sepsis | 0.674 | 0.774 | 1899 | 0 |
|  | CD44 | Control | 0.565 | 0.82 | 3877 | 0 |
|  | EXT1 | Sepsis | 0.017 | 0.135 | 1899 | 0.0222 |
|  | EXT1 | Control | 0.012 | 0.136 | 3877 | 0.0222 |
|  | EXT2 | Sepsis | 0.056 | 0.25 | 1899 | 0.00045 |
|  | EXT2 | Control | 0.037 | 0.225 | 3877 | 0.00045 |
|  | HIF1A | Sepsis | 0.215 | 0.474 | 1899 | 0 |
|  | HIF1A | Control | 0.16 | 0.475 | 3877 | 0 |
|  | HMMR | Sepsis | 0.005 | 0.083 | 1899 | 0.02527 |
|  | HMMR | Control | 0.002 | 0.052 | 3877 | 0.02527 |
|  | SELL | Sepsis | 1.017 | 1.02 | 1899 | 0.00193 |
|  | SELL | Control | 0.93 | 1.039 | 3877 | 0.00193 |
| NK cells | CD44 | Sepsis | 0.321 | 0.654 | 831 | 0.20119 |
|  | CD44 | Control | 0.3 | 0.663 | 1254 | 0.20119 |
|  | EXT1 | Sepsis | 0.013 | 0.122 | 831 | 0.59726 |
|  | EXT1 | Control | 0.012 | 0.123 | 1254 | 0.59726 |
|  | EXT2 | Sepsis | 0.08 | 0.314 | 831 | 0.08558 |
|  | EXT2 | Control | 0.065 | 0.3 | 1254 | 0.08558 |
|  | HIF1A | Sepsis | 0.194 | 0.503 | 831 | 0.01833 |
|  | HIF1A | Control | 0.15 | 0.451 | 1254 | 0.01833 |
|  | HMMR | Sepsis | 0.018 | 0.141 | 831 | 0.00099 |
|  | HMMR | Control | 0.005 | 0.085 | 1254 | 0.00099 |
|  | SELL | Sepsis | 0.537 | 0.9 | 831 | 0.00051 |
|  | SELL | Control | 0.433 | 0.896 | 1254 | 0.00051 |
| B cells | CD44 | Sepsis | 0.357 | 0.522 | 361 | 0.00445 |
|  | CD44 | Control | 0.334 | 0.727 | 366 | 0.00445 |
|  | EXT1 | Sepsis | 0.016 | 0.113 | 361 | 0.38802 |
|  | EXT1 | Control | 0.009 | 0.08 | 366 | 0.38802 |
|  | EXT2 | Sepsis | 0.036 | 0.165 | 361 | 0.02818 |
|  | EXT2 | Control | 0.018 | 0.127 | 366 | 0.02818 |
|  | HIF1A | Sepsis | 0.199 | 0.503 | 361 | 0.07206 |
|  | HIF1A | Control | 0.186 | 0.572 | 366 | 0.07206 |
|  | HMMR | Sepsis | 0.093 | 0.265 | 361 | 0 |
|  | HMMR | Control | 0.028 | 0.171 | 366 | 0 |
|  | SELL | Sepsis | 0.589 | 0.864 | 361 | 0.16609 |
|  | SELL | Control | 0.568 | 0.953 | 366 | 0.16609 |
| HSC | CD44 | Sepsis | 0.393 | 1.02 | 3446 | 0 |
|  | CD44 | Control | 0.155 | 0.757 | 1955 | 0 |
|  | EXT1 | Sepsis | 0.016 | 0.211 | 3446 | 0.01171 |
|  | EXT1 | Control | 0.005 | 0.141 | 1955 | 0.01171 |
|  | EXT2 | Sepsis | 0.004 | 0.135 | 3446 | 0.451 |
|  | EXT2 | Control | 0.002 | 0.1 | 1955 | 0.451 |
|  | HIF1A | Sepsis | 0.347 | 0.958 | 3446 | 0 |
|  | HIF1A | Control | 0.117 | 0.662 | 1955 | 0 |
|  | HMMR | Sepsis | 0 | 0 | 3446 | NA |
|  | HMMR | Control | 0 | 0 | 1955 | NA |
|  | SELL | Sepsis | 0.441 | 1.11 | 3446 | 0 |
|  | SELL | Control | 0.235 | 0.955 | 1955 | 0 |
| Neutrophils | CD44 | Sepsis | 0.136 | 0.745 | 1818 | 0.05403 |
|  | CD44 | Control | 0.087 | 0.619 | 950 | 0.05403 |
|  | EXT1 | Sepsis | 0.009 | 0.199 | 1818 | 0.14813 |
|  | EXT1 | Control | 0 | 0 | 950 | 0.14813 |
|  | EXT2 | Sepsis | 0 | 0 | 1818 | NA |
|  | EXT2 | Control | 0 | 0 | 950 | NA |
|  | HIF1A | Sepsis | 0.316 | 1.12 | 1818 | 0.0064 |
|  | HIF1A | Control | 0.212 | 0.958 | 950 | 0.0064 |
|  | HMMR | Sepsis | 0 | 0 | 1818 | NA |
|  | HMMR | Control | 0 | 0 | 950 | NA |
|  | SELL | Sepsis | 0.656 | 1.545 | 1818 | 0.00262 |
|  | SELL | Control | 0.488 | 1.428 | 950 | 0.00262 |
