# Supplementary material for: Identification and validation of glycosylation-related gene signatures for prognostic stratification in sepsis
Source: Front Immunol. 2025 Jul 2;16:1608082. doi: 10.3389/fimmu.2025.1608082 (PMC12263689; doi:10.3389/fimmu.2025.1608082)
Supplement: Supplementary file 10 [file Table6.docx]

Supplementary Table 6. Baseline of patient characteritics

| Variables | Total (n=50) | Healthy (n=20) | Sepsis (n=30) | P value | SMD |
| --- | --- | --- | --- | --- | --- |
| Survival status |  |  |  | 0.003 | 0.333 |
| survival | 40 (80%) | 20 (100%) | 20 (67%) |  |  |
| non-survival | 10 (20%) | 0 (0%) | 10 (33%) |  |  |
| Age, mean ± SD | 45.02 ± 14.64 | 42.85 ± 13.13 | 46.47 ± 15.62 | 0.381 | 0.251 |
| Gender, n (%) |  |  |  | 0.345 | 0.000 |
| Female | 15 (30%) | 8 (40%) | 7 (23%) |  |  |
| Male | 35 (70%) | 12 (60%) | 23 (77%) |  |  |
| SOFA, median (Q1,Q3) | 2 (0, 6) | 0 (0, 0) | 5 (3, 9) | <0.001 | 2.446 |
| Foci of infection |  |  |  | <0.001 | NA |
| Non | 20 (100%) | 20 (100%) | 0 (0%) |  |  |
| Lung | 12 (24%) |  | 12 (40%) |  |  |
| Blood | 9 (18%) |  | 9 (30%) |  |  |
| Others | 9 (18%) |  | 9 (30%) |  |  |
| Culture, n (%) |  |  |  | <0.001 | 1.061 |
| Non | 20 (100%) | 20 (100%) | 0 (0%) |  |  |
| G- | 24 (48%) |  | 24 (80%) |  |  |
| G+ | 5 (10%) |  | 5 (17%) |  |  |
| Others | 1 (2%) |  | 1 (3%) |  |  |
